# Development and psychometric evaluation of scales to measure professional confidence in manual medicine: a Rasch measurement approach

**DOI:** 10.1186/1756-0500-7-338

**Published:** 2014-06-04

**Authors:** Mark D Hecimovich, Irene Styles, Simone E Volet

**Affiliations:** 1 School of Psychology and Exercise Science, Murdoch University, South Street, Murdoch 6150, Western Australia; 2 Graduate School of Education and Pearson Psychometric Centre, The University of Western Australia, Crawley 6009, Western Australia; 3 School of Education, Murdoch University, South Street, Murdoch 6150, Western Australia

**Keywords:** Confidence, Communication, Clinical skills, Rasch measurement model, Manual medicine

## Abstract

**Background:**

Health professionals in athletic training, chiropractic, osteopathy, and physiotherapy fields, require high-level knowledge and skills in their assessment and management of patients. This is important when communicating with patients and applying a range of manual procedures. Prior to embarking on professional practice, it is imperative to acquire optimal situation-specific levels of self-confidence for a beginner practitioner in these areas. In order to foster this professional self-confidence within the higher education context, it is necessary to have valid and reliable scales that can measure and track levels and how they change. This study reports on the development and psychometric analysis of two new scales, Patient Communication Confidence Scale (PCCS) and the Clinical Skills Confidence Scale (CSCS), to measure confidence in these two areas for students in manual medicine programs. The Rasch measurement model was used to guide the development of the scales and establish their psychometric properties.

**Methods:**

The responses to 269 returned questionnaires over two occasions were submitted to psychometric analysis, with various aspects of the scales examined including: item thresholds; item fit; Differential Item Functioning; targeting; item locations; item dependencies; and reliability. To provide further evidence of validity, scores were correlated with two existing valid scales.

**Results:**

Analyses showed that the scales provided valid and reliable measures of confidence for this sample of persons. High Person Separation Indices (0.96 for PCCS; 0.93 for SCSC) provided statistical evidence of reliability, meaning the scales are able to discriminate amongst persons with different levels of confidence. For the PCCS, item categories were operating as required, and for the CSCS only two items’ thresholds were slightly disordered. Three tests of fit revealed good fit to the model (indicating the internal consistency of both scales) and results of the correlations with two existing valid scales were consistent with expectations.

**Conclusions:**

The importance of confidence cannot be overlooked in health education because students learning new information and skills, and dealing with challenging situations can be negatively impacted by a lack of confidence which can result in students disengaging from placements or leaving a program. Valid and reliable instruments are essential in tracking change in levels of confidence in specific skills over time and the examination of the degree of congruence between confidence and competence. Analysis of responses to the two confidence scales established that they are valid and reliable instruments.

## Background

Health professionals in athletic training, chiropractic, osteopathy, and physiotherapy fields, require high-level knowledge and skills in their assessment and management of patients. This is particularly important when communicating with patients and applying a range of manual procedures, such as physically assessing joints and soft-tissue, performing manipulative or soft-tissue procedures, carrying out rehabilitation protocols, and bracing and taping techniques. Prior to embarking on professional practice, it is imperative to acquire optimal situation-specific levels of self-confidence for a beginner practitioner in these areas. In order to purposely foster this professional self-confidence within the higher education context, it is advantageous to have valid and reliable scales that can measure and track levels and how they change, and that may be used in conjunction with objective measures of competence.

Although findings on the congruence between self-confidence and competence in health education are mixed [1-5], what is clear from previous research is that patients assessed and treated by providers with an appropriate level of confidence in these skills often have better outcome expectancies [6,7]. Therefore, for students undertaking internships in manual medicine programs such as athletic training, chiropractic, osteopathy and physiotherapy, the development of scales to measure their confidence in these skills is crucial. Assessing professional confidence is also important because both sub-optimal and above-optimal levels of confidence can negatively impact on learning new skills and dealing with challenging situations. For example, under-confident students may visualise defeat before it occurs [8], which can lead to decreased competence, whereas over-confident students may feel competent in procedures without having prior clinical experience [9], which can be potentially dangerous for patients. A critical review of literature shows that self-confidence is referred to and utilised in a variety of contexts, from leadership skill sets to athleticism to skill acquisition for trainee health care providers [10]. Self-confidence is a self-construct or individual characteristic that enables a person to have a positive or credible view of themselves, in situations or tasks they encounter [11]. It refers to a person’s expectation of his or her ability to achieve a desired goal or outcome in a given locale, and is a highly influential factor in determining an individual’s potential [12]. In other words, a person with high self-confidence has an assured view of themselves and their capabilities, their knowledge and skills, which contribute to making them persist in an endeavour and help others form an impression of a credible professional.

In an education context, both high and low achievement students are typically informed and guided by their beliefs and perceptions rather than by reality [13]; in other words, by their level of self-confidence about what they believe they are capable of. This pertains more to new endeavours as opposed to experiences previously encountered. For example, Koriat et al. [14], argue two primary reasons for self-confidence. First, one assesses his or her knowledge and skill of a situation or task, which includes previous experience. Second, based on that evidence, the situation is reviewed and a belief-level is chosen about how successful one feels he will be. In health professional education programs, educators, supervisors and mentors need to be aware of this construct and tendency, and purposely promote student self-confidence in order to avert or reverse a negative mindset. Additionally, they need to be able to measure levels of self-confidence and improve low levels and, in comparison with objective measures of competence, address issues of misplaced over-confidence. Bandura’s [15] social learning theory posits motivation, reinforcement and past experience as key components that promote self-confidence. Promoting self-confidence early in clinical training provides a crucial foundation for the successful acquisition and implementation of vital knowledge and skills [8]. Several authors note the more clinical successes a student experiences the more self-confidence is reinforced [16-20].

Given that professional confidence is important, health educators need to know what learning activities aid its development, and have appropriate tools to measure any change [21]. The value of developing valid and reliable measures of students’ confidence in clinical and patient communication skills during their studies is threefold: first, to track changes in levels of confidence in specific skills over time; second, to examine the degree of congruence between confidence and competence; and third, to identify over-confident and under-confident students.

However, the availability of psychometrically robust instruments to measure professional confidence in manual medicine education is limited. Therefore, this paper describes a research study which aimed to develop two new scales to measure confidence in clinical and patient communication skills for students in manual medicine programs, and to use the Rasch measurement model [22] to establish the psychometric properties of these scales. The paper focuses on these issues rather than the presentation of substantive results from the study. The aims in regard to the psychometric properties of the scales were checking (a) the internal consistency (including the possibility of sub-scales) and reliability of the items; (b) the invariance of the operation of items across persons from different designated groups (known as differential item functioning (DIF)); and (c) the convergent validity of the new scales by examining the correlations between the two new scales and two existing scales which are related to, but different from, confidence. Whilst the Rasch paradigm is being increasingly used in the development and evaluation of clinical tools in health and medical sciences, including rehabilitation science, psychology, nursing and podiatry [23-26], it is relatively novel in manual medicine research. The scales presented in this paper were primarily designed for chiropractic students, but they could be adapted for use with athletic training, physiotherapy, and osteopathic students.

Overall, the aims of this study were to develop and validate measurement tools for use in health education and research. It is innovative in that it recognises the vital link between confidence in communication as well as clinical skills and patient outcomes, thus the development of two separate scales was deemed important. The use of the Rasch model adds to the novel aspects of the paper.

## Method

### Development of scales

The data garnered from various informal student group interviews and critical reviews of literature helped shape the content and objectives of the self-confidence scales and eventual confidence questionnaire. Items and their format were developed after careful review of related scales [27-30] and health education studies, which demonstrated different ways to assess professional self-confidence in educational or clinical internship settings [21,31-34]. Whilst these studies did not mirror the aims of the present research, they provided vital evidence regarding the importance of professional self-confidence in health education programs. A few items were also developed based on one of the authors’ extensive experience in athletic training, chiropractic and physical therapy curricula. They were those focussing on the ability to discuss health risk behaviours (diet, drug use, and exercise), application of orthopaedic bracing, supports and taping, and demonstrating rehabilitative procedures.

Accordingly, a preliminary instrument of 52 items or statements was developed with a primary focus on patient communication and clinical skills, and (due to their role in self-confidence, which was identified through the informal interviews and critical review of literature) a secondary focus on supervising clinicians. A six-point Likert-style response format for each item was utilised. Response categories were coded from 1 (“not confident at all”) to 6 (“very confident”). The questions reflected interactions and experiences with patients that students were likely to encounter, and ranged from discussing general health issues to performing basic and focused physical examination procedures. For example: “How confident are you in your ability to discuss personal and/or sensitive issues with new patients?”; and “How confident are you in your ability to perform basic physical examination procedures such as blood pressure, pulse and respiration rate on a patient?” Content validity was assessed by a panel of educators and researchers affiliated with education programs and chiropractic clinical education internship programs in Australia and the United States. Panel members were asked to review the scale and comment on each item and the overall format. They suggested minor alterations for a few items, and recommended the inclusion of a demographic section and a self-reflection section that invited the students to qualify their responses.

The scale was divided into two parts. One part of the scale focussed on patient communication, labelled the Patient Communication Confidence Scale (PCCS). The other part focussed on clinical skills, labelled the Clinical Skills Confidence Scale (CSCS). The two scales represent different aspects of self-confidence, both of which are important, and more diagnostic information about these two different aspects could be gained by measuring them separately and thus being able to see whether levels differ. If levels on the two aspects were to differ, different teaching strategies could be aimed at developing each aspect. It is an empirical question whether the two scales could be conceived as representing the same construct and for some purposes a single score may be all that is required to make teaching and learning decisions. However, in this case, the research aimed to get information about each of the two aspects as they are each essential and are likely to require different strategies to address them.

To assist with the validation process, two existing valid and reliable scales were incorporated, the Personal Report of Communication Apprehension (PRCA-24) and General Self-efficacy (GSE) scales. The PRCA-24 scale measures feelings about communicating with others. However, only one sub-category (interpersonal communication) was used in this study, as the other sub-categories are not typically encountered in clinical contexts. Prior research has demonstrated content, criterion, and construct validity of the PRCA-24 [35]. The GSE scale was added to gather data regarding the generalised self-efficacy of the students, and to compare their general self-efficacy and specific task-related self-efficacy measures. Previous research shows the GSE is a reliable scale with convergent and discriminant validity, with alpha reliability coefficients ranging from .75 to .90 [36,37]. It was expected that the PCCS and CSCS would correlate positively with the GSE and negatively with the PRCA-24 Interpersonal communication sub-scale; however, these correlations were not expected to be very high because the PRCA-24 and the GSE are designed to assess constructs that are similar but not identical to self-confidence. The final confidence questionnaire (CQ) contained the following:

1. General Self-efficacy Scale (GSE).

2. Personal Report of Communication Apprehension Scale (PRCA-24), Interpersonal communication sub-scale.

3. Patient Communication Confidence Scale (PCCS), 28 items, 6 response categories, no reverse items.

4. Clinical Skills Confidence Scale (CSCS), 27 items, 6 response categories, no reverse items.

5. Self-reflection section.

6. Demographic section.

The PCCS items covered nine aspects of patient communication such as encouraging behaviour change, history-taking, explaining, and being supportive. The CSCS addressed eight aspects of confidence in clinical skills such as manipulative, X-ray, and physical examination procedures.

### Participants and questionnaire administration

Participants included seven cohorts of chiropractic students (n = 269) enrolled in internships in tertiary institutions in Australia and the United States. All cohorts had comparable clinical curricula that provided similar professional experiences such as recording patient histories, and supervised assessment and treatment of patients. Human ethics approval and student consent were obtained. The CQ was administered at the beginning of students’ clinical internships and was repeated five months later (one cohort—it was not feasible to retest all cohorts at this time) and again ten months later (all cohorts). Data from only the first and third occasions (beginning of the study and ten months later) were used to examine the validity and reliability of the PCCS and CSCS. Combining data in this way is an accepted procedure made possible by the Rasch model’s properties of invariant comparisons. The legitimacy of the procedure can be tested empirically using differential item functioning (DIF).

### Data analysis

Student responses to 269 returned questionnaires over two occasions were submitted to psychometric analysis using the polytomous Rasch model (PRM) [22,38], through the Rasch Unidimensional Measurement Model software RUMM2030 [39]. This model was used to establish whether the two new scales had been operationalised successfully, and to appraise aspects of the validity and reliability of the scales [40]. The Rasch model was selected because it is the only measurement model in the social sciences that has the desirable scaling properties of invariance of comparisons [41-44]. The model requires that a comparison between any two persons from a given class of persons should be independent of which items in a given class of items are chosen for the comparison, and the comparison of any two items from a given class of items should be independent of which persons in a given class of persons are chosen to make the comparison [45]. For more detailed explanations of the Rasch paradigm and procedures, see, for example, Andrich [41], Andrich and Styles [40], Bond and Fox [43] and the online manual for the RUMM2030 software [40]. For many researchers, the Rasch paradigm represents an advance on classical test theory [40,43,46,47]. In both theories, for example, the total score of a person on an instrument is the relevant statistic to represent a person’s standing on the variable or property of interest. However, the raw scores used in classical test theory are not linearised (they are linearised in Rasch measurement) and should not be treated as measurements.

The Rasch model can be used to examine data for flaws or problems indicated by a failure to fit the model [48]. Showing that an item’s responses (data) fit the model is shorthand for concluding that the item operates consistently with the other items in a scale to characterise a single variable as summarised by the Rasch model. Therefore, if responses to a set of items in a scale fit the Rasch model, they are established as being internally consistent—which is a prerequisite for confirming construct validity. Further, measures for persons may then be legitimately used in basic mathematical operations (such as addition) and thus subjected to standard statistical procedures. Two important properties are present if data fit the model: first, the measures of participants will be on a *linear scale*; and second, the measures will be *invariant* (the relative ordering of items and persons will be the same no matter which items are used to compare persons, and no matter which persons are used to compare items). In addition, examination of differential item functioning will provide evidence of whether measures are invariant (essentially, whether they represent the same construct) across designated groups for which the fit has been confirmed [40,41,46].

In the Rasch model, the relevant statistic for any person is simply the total score across items where the scores are successive integers assigned to successive categories, which is the same statistic as that used traditionally. Some items may be dichotomous, and some may have more than two ordered categories. However, these scores are not themselves linear and should not generally be treated as measurements. In particular, they are affected by floor and ceiling effects so that a difference of a raw score of 2, say, at one part of the continuum of the construct does not represent the same difference as a score of 2 on another part of the continuum. The transformation of the raw scores using the Rasch model produces linearised scores for each person which can be treated as measurements and used in standard statistical analyses. These linearised scores are known as *locations*. More formally, the Rasch model provides measurements that are compatible with fundamental or additive conjoint measurement studied in mathematical psychology [40].

The Rasch model is a probabilistic one which provides an appropriate model for typical social science data. For polytomous items the equation takes the form:

(1) $$\mathrm{Ρr}\left\{X_{\mathrm{ni},}=x;\beta_{n},\left({\delta_{i}}_{k}\right)\right\}=\left[\exp\left(x\beta_{n}-\sum_{k=0}^{x}\delta_{\mathrm{ik}}\right)\right]/\gamma_{\mathrm{ni}}$$

where (i) *X*_*ni*_, is the random variable of the response of person *n* to item *i* and where the value of this variable is an integer 0, 1, 2, 3, …, *m*, *β*_*n*_ is the location of the person on the variable, (*δ*_*ik*_), *k* = 1, 2, 3, …, *m*_*i*_ is a vector of thresholds of item *i* at which the probability of a response in adjacent categories is identical, and $\gamma_{\mathrm{ni}}=\sum_{x=0}^{m}\left[\exp\left(x\beta_{n}-\sum_{k=0}^{x}\delta_{\mathrm{ik}}\right)\right]$ is the sum of the numerators and ensures that Eq. (1) sums to 1 and is a probability statement [49,50].

The RUMM2030 software [39] provides an extensive range of facilities for assessing the quality of items in a scale. Facilities include several different statistical (chi square and log residual tests of fit) and graphical tests of fit (Category and Item Characteristic Curves) between the data and the model, and an index of reliability, known as the Person Separation Index (PSI). The program also provides information on the targeting of person and items (whether the spread of item and person locations are similar), and on item dependencies and the possibility of meaningful sub-scales through residual item correlations, residual principal component analysis, and sub-scale analysis. In combination, this information is used to ascertain the quality of a scale and to identify anomalies in the data, which may lead to a deeper understanding of the construct or property being measured.

As mentioned in the introduction, data analysis addressed three primary aims, the first of which was to establish the internal consistency and reliability of each scale. In other words, do the sets of items each represent a single construct at this level of scale? If they do, then one is justified in adding scores to obtain a total score on each scale and then using those total scores (or their linearised equivalents known as locations) for other statistical tests such as comparisons of mean scores amongst groups or over time.

The second aim was to determine whether the items of each scale have the same psychometric properties across different groups of participants: this is termed Differential Item Functioning (DIF) and it determines whether the items have similar psychometric properties across different groups of participants, that is, whether the items have invariant properties across groups. If items show DIF across groups, they should not be used to compare person performance, unless individuals are from the same group. In this study, the groups of interest were gender, age, experience with the profession, entry qualification (previous degree or not) and the occasion of administration.

The third aim was to provide evidence of the convergent validity of the PCCS and CSCS by examining their statistical correlations with the established GSE and PRCA-24 scales which assess some aspects of confidence.

To address the first aim, various aspects of the scales were examined. The first aspect was the operation of the response categories. The item thresholds (the cut-points between each successive pair of categories such as, Strongly Agree and Agree) are required to be correctly ordered. The second aspect was the fit of each set of items to the Rasch model. If the items fit the model, which is evidence of internal consistency, they can be accepted as measuring a single variable at this level of scale. Two tests of fit - one statistical (the chi square) and one graphical (the Item Characteristic Curves, ICCs) - were used to judge this. In the Rasch paradigm generally, no one test of fit is sufficient to make a decision about fit. A third aspect was the targeting of items and persons to each other: this is established by examining the joint distribution of item and person locations on the same continuum. A fourth aspect, item dependencies, was examined by inspection of the residual correlations between items. If items show dependency, then one item in each pair is redundant and retaining both artificially increases the reliability. Such dependencies may also indicate the presence of sub-scales which can be further examined through the principal component analysis of residuals. Lastly, reliability is gauged using the Person Separation Index (PSI), which is the Rasch equivalent of Cronbach’s alpha.

To address the second aim – to establish whether the items operate relatively consistently across different groups, differential item functioning across the groups for Gender, Age, Prior Experience, Entry qualification and Occasion was examined.

Lastly, to address the third aim to provide further evidence of validity (this time, convergent validity), student scores on the scales were correlated with scores from the same students on two existing scales that measure constructs related to but different from confidence and whose validity has been established in the research literature, namely, the GSE and PRCA-24 (interpersonal communication).

The results of these analyses provide information about the validity and reliability of the two scales. If these are satisfactory, the person locations (the linearised raw scores) can be used for further analyses as, for example, the comparison of mean scores (person locations) for the different groups of interest, and the investigation of changes in mean locations over time.

## Results

Two hundred and sixty-nine students agreed to complete the questionnaire (269 matched questionnaires across two occasions) with the following demographics: 153 males and 116 females; age: 20–25 years (136), 26–35 years (106), 36+ years (27); possession of degree upon entry, 153 students, no degree upon entry, 116 students.

Using complete information for each person and item, the scales were analysed using the polytomous Rasch model with 10 possible response categories. Table 1 provides a summary of the results of the Rasch analyses, and subsequent sections address different analytical aspects in more detail.

**Table 1:** Summary of Rasch analyses for the PCCS and CSCS with DIF according to Gender (Gen), Age, Experience (Exp), Entry qualification (Ent) and Occasion of Administration (Occ)

**Scale**	**Disorder-ed items**	**Significant residual correlations (>0.3)**	**Misfitting items**	**DIF**					PSI*
				**Gen**	**Age**	**Exp**	**Ent**	**Occ**	
PCCS	none	18 pairs	20, 16	none	none	none	none	none	0.962
CSCS	12, 27	20 pairs	1, 13	none	none	none	none	none	0.933

*Person Separation Index.

Analysis of the operation of the categories for the PCCS (28 items) and CSCS (27 items) indicated two items having disordered categories in the CSCS and none in the PCCS. There were also several significant residual correlations between pairs of items in both scales. The three tests of fit - two statistical (log-residual and item-trait interaction) and one graphical (the Item Characteristic Curves, ICCs) – revealed two misfitting items each in the PCCS and CSCS. No items in either scale showed Differential Item Functioning (DIF) according to any of the groups of interest. Although the Person Separation Indexes (PSI) were high at 0.962 (PCCS) and 0.933 (CSCS), these results are likely to have been inflated due to the high number of item dependencies as indicated by significant residual inter-item correlations.

Upon review of the initial analysis, simultaneous examination of several aspects of each item was undertaken in order to achieve an improved psychometric properties for each scale. This examination included analysis of the operation of item thresholds (the cut points between adjacent categories), item fit, the item’s location, item dependencies (residual inter-item correlations), DIF, and the targeting between items and persons). Items that were flagged as anomalous by the statistics were examined for wording and content. If any plausible issue was detected with the item, it was removed from the scale.

The following section addresses different aspects of the analytical review process and actions that were taken to improve them where necessary.

### Item thresholds

For the PCCS, the six response categories operated as required, with all items having ordered thresholds. For the CSCS, two items (12 and 27) had slightly disordered thresholds, meaning that the categories were not operating correctly. The number of categories for item 12 was reduced to three (0, 1 and 2), and those for item 27 reduced to four (0, 1, 2 and 3). Re-analysis then indicated that the categories for all items were ordered correctly.

### Item fit

For each scale, two tests of fit were considered - the item-trait interaction (chi square) statistical test, and the Item Characteristic Curve (ICC) graphical test. These revealed generally good fit to the model. The results of the statistical tests of fit for the initial analyses with all items are shown in Table 2 and Table 3 (Bonferroni-adjusted probability values were used for the chi square test).

**Table 2:** Locations (in increasing order) and Chi square fit statistics for the PCSC

**Item**	**Location**	**Chi Sq**	**Probability**	**Item content**
PC22	−1.458	19.922	0.001	*Interviewing patient of same gender*
PC25	−1.453	20.383	0.001	*Interviewing patient of similar age*
PC2	−1.150	6.036	0.302	*Introducing self to new patient*
PC23	−1.082	7.681	0.175	*Interviewing a teenage patient*
PC21	−1.054	19.138	0.002	*Interviewing a patient of the opposite gender*
PC8	−0.959	6.250	0.283	*Taking new patient’s health history*
PC3	−0.925	7.349	0.196	*Starting conversation with new patient*
PC24	−0.774	11.295	0.046	*Interviewing a patient 60+ years of age*
PC7	−0.365	16.935	0.005	*Expressing empathy in an interview*
PC1	−0.361	9.312	0.097	*Overall confidence in patient communication*
PC5	−0.343	1.340	0.931	*Using non-verbal interactions*
PC16	−0.145	24.808	0.000	*Explaining segmental joint dysfunction*
PC10	−0.126	6.209	0.286	*Discussing personal issues with current patients*
PC12	−0.096	2.585	0.764	*Discussing preventative health strategies*
PC11	0.160	2.702	0.746	*Discussing health risk behaviours*
PC4	0.183	2.964	0.706	*Discussing changes in treatment with current patients*
PC28	0.350	10.006	0.075	*Discussing with patient whilst being observed by lower level peer*
PC20	0.380	37.409	0.000	*Interviewing wheelchair-bound patient*
PC15	0.382	9.804	0.081	*Obtaining informed consent*
PC13	0.397	5.252	0.386	*Encouraging patients to improve health habits*
PC6	0.417	3.066	0.690	*Asking non-scripted open-ended questions*
PC17	0.649	8.864	0.115	*Telling a patient you may not be able to help them*
PC9	0.678	10.375	0.065	*Discussing personal issues with new patients*
PC26	0.855	19.72	0.001	*Communicating with patients after failed manipulation*
PC27	0.995	10.969	0.052	*Interviewing new patient whilst being observed by lower level peer*
PC14	1.507	2.510	0.775	*Telling people they have a serious condition*
PC18	1.586	4.380	0.496	*Counselling a distraught patient*
PC19	1.751	2.323	0.803	*Dealing with a disruptive patient*

**Table 3:** Locations (in increasing order) and Chi square fit statistics for the CSCS

**Item**	**Location**	**Chi Sq**	**Probability**	**Item content**
CS23	−1.580	4.878	0.431	*Manipulating new patient while being observed by clinician you are comfortable with*
CS25	−1.313	4.248	0.514	*Manipulating current patient while being observed by clinician you are comfortable with*
Cs2	−1.306	4.319	0.505	*Basic physical exam procedures (B/P)*
CS3	−1.157	5.417	0.367	*General physical exam procedures*
CS11	−1.083	2.907	0.714	*Performing soft-tissue procedures*
CS9	−0.999	9.785	0.082	*Performing spinal manipulation*
CS1	−0.997	47.743	0.000	*Overall confidence in clinical skills*
CS6	−0.992	28.861	0.000	*Focused spinal musculoskeletal examination*
CS8	−0.733	37.778	0.000	*Assessing soft tissue and joint lesions*
CS5	−0.708	5.140	0.399	*General musculoskeletal examination*
CS17	−0.163	13.267	0.021	*Administering modalities (US, EMS)*
CS14	−0.110	9.836	0.080	*Demonstrating rehabilitative procedures*
CS20	0.084	9.480	0.091	*Using one manipulative technique*
CS7	0.116	25.133	0.000	*Focused extremity musculoskeletal examination*
CS15	0.235	3.166	0.675	*Positioning patient for spinal x-ray*
CS21	0.441	28.834	0.000	*Using two manipulative techniques*
CS4	0.450	3.139	0.679	*Detailed physical exam procedures (CV, GIT)*
CS16	0.483	1.992	0.850	*Positioning patient for extremity x-ray*
CS10	0.675	7.679	0.175	*Performing extremity manipulation*
CS27	0.735	8.647	0.124	*Performing manipulation after previously failed attempt*
CS12	0.762	97.615	0.000	*Taping techniques*
CS22	0.855	17.356	0.004	*Using three + manipulative techniques*
CS24	1.180	6.095	0.297	*Manipulating new patient while being observed by clinician you are uncomfortable with*
CS26	1.183	8.938	0.112	*Manipulating current patient while being observed by clinician you are uncomfortable with*
CS13	1.206	22.971	0.000	*Appling bracing/supports*
CS18	1.311	8.306	0.140	*Manipulating pregnant patient*
CS19	1.428	4.780	0.443	*Manipulating wheelchair bound patient*

**Figure 1:**
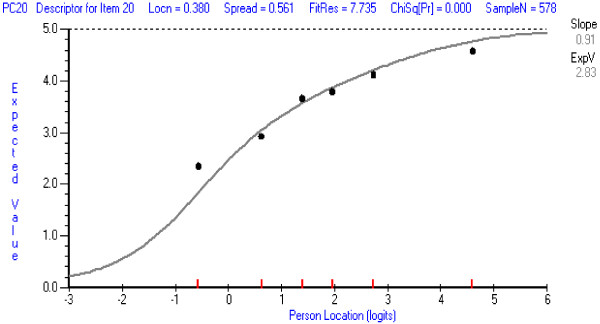
**Item Characteristic Item Curve for PCCS item with least good fit: Item 20 *Interviewing a wheelchair-bound patient.***

**Figure 2:**
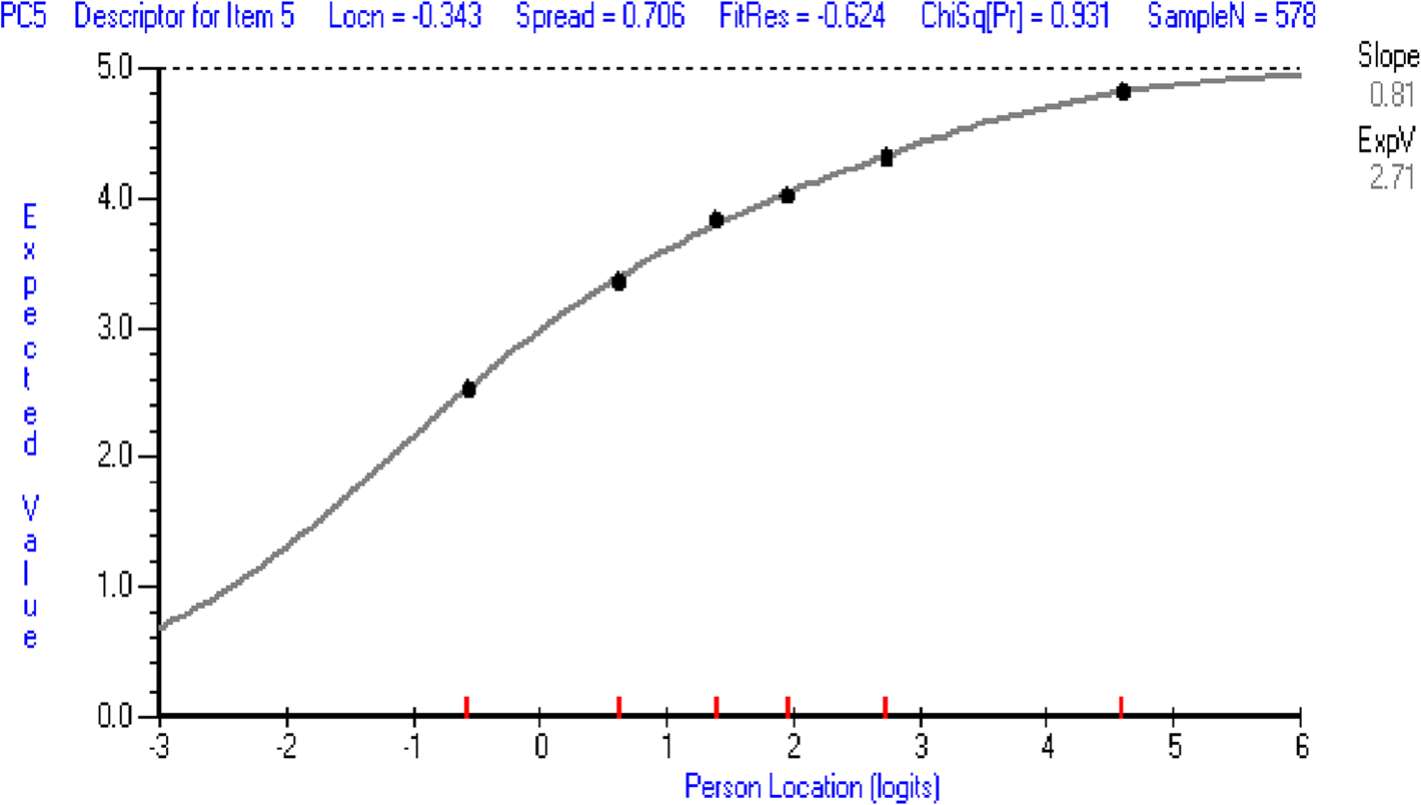
**Item Characteristic Item Curve for a PCCS item with good fit: Item 5 *Using non-verbal interactions.***

In the PCCS, item 20 (*confidence in taking a medical history/interviewing a patient who is wheelchair bound*) was the least well-fitting according to the chi square statistical test and the graphical test (ICC) of fit. The ICCs for this item and for an item with good fit (for comparison), are shown in Figures 1 and 2.

The ICCs show the theoretical probabilities (the continuous curve) of endorsing the item across the range of person locations on the whole set of PCCS items, and the obtained probabilities for six class-intervals of person locations (the dots). The dots should follow the theoretical curve closely if fit is good. The chi square statistic represents the deviations of the obtained dots from the theoretical curve. The operation of Item 20 is a little inconsistent and tends not to discriminate as well as the other items amongst persons with different total scores particularly at the highest and lowest person locations (the obtained dots are, respectively, below and above the theoretical curve). This may be because it addresses a very specific situation which students may or may not have encountered. When this item was deleted and the PCCS re-analysed, only item 16 (*confidence in explaining segmental joint dysfunction*) showed some minor misfit. However, all items could be retained at present because Item 20 represents an important aspect of clinical practice, represents less than 5 percent of all items, and is unlikely to have a marked effect on person measures. Its performance should be monitored in future analyses. Item 16 (confidence in *explaining segmental joint dysfunction*), although appearing to be more chiropractic or osteopathic orientated, represents an important component which is discussed with patients who may undergo manipulative procedures by various manual health care professional such as physical therapists, and athletic trainers. Therefore it too should be retained for future evaluation.

**Figure 3:**
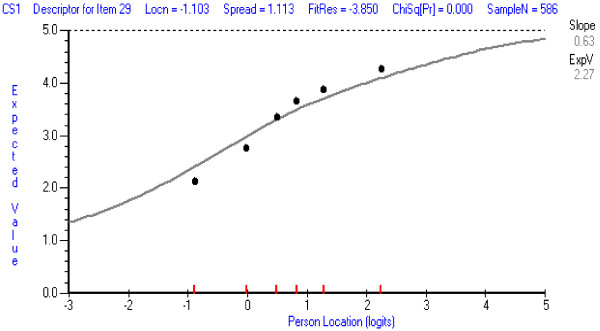
**Item Characteristic Item Curve for CSCS item with least good fit: Item 1 *Overall confidence in clinical skills.***

**Figure 4:**
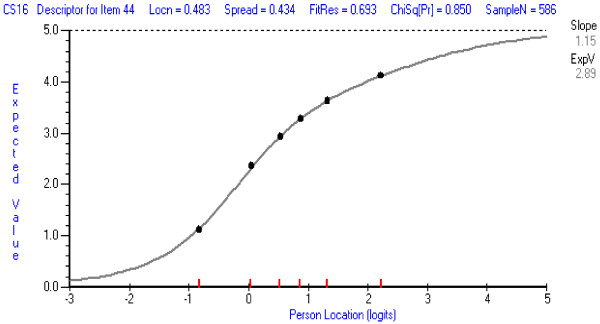
**Item Characteristic Item Curve for a CSCS item with good fit: Item 16: *Position patient for extremity x-ray.***

In the CSCS, item 1 (*general overall level of confidence in the area of application of clinical skills*) was the least well-fitting item, and was deleted from further analysis. It tended to over-discriminate—a typical result for items that are aimed at assessing a property in an overall or general sense. The ICCs for this item and for an item with good fit are shown in Figures 3 and 4. When the CSCS was re-analysed, only item 13 showed some misfit (*confidence in ability to apply orthopaedic bracing or supports on a patient*). It tended not to discriminate as well as other items, but this item was retained at this stage because it refers to a commonly used therapeutic procedure in the scale’s targeted population. Its performance should be reassessed in future analyses.

### Item locations

Tables 2 and 3 also show the locations of items (in logits, the Rasch “unit” of measurement) with items listed according to increasing intensity. The lowest locations indicate the easiest items to agree with, meaning that even participants with relatively low levels of confidence are likely to agree with these. Conversely, the highest locations indicate the items that require relatively high levels of confidence to agree with. For the PCCS, item 22 was the easiest (*confidence in ability at taking a medical history/interviewing a patient of the same gender*), and item 19 was the most difficult (*confidence in dealing with ‘disruptive’ patients*). For the CSCS, item 24 was the easiest (*confidence in ability to perform spinal manipulative procedures on a new patient while being observed by a clinician whom you who are comfortable with*), and item 27 was the most difficult (*confidence in ability to perform spinal manipulative procedures on a patient who had been successfully manipulated by a clinician after a failed attempt by you*). These results were as expected theoretically, in terms of the relative levels of confidence needed to agree with each item. This ordering of items provides evidence of the construct validity of the scales.

### Item dependencies

In the PCCS and CSCS, respectively, 18 and 20 pairs of items showed dependencies (residual correlations of >0.3). This means that one item of each pair is not adding much information about levels of confidence, and, further, these dependencies are artificially inflating the reliabilities. For example, one of the two items 3 and 8 (*confidence in ability to start a conversation with a new patient* and *confidence in ability at taking a new patient’s current and past health medical history*) is redundant. Accordingly, the PSIs for the PCCS (0.962) and CSCS (0.930) are likely to be inflated, but nevertheless suggest the scales can provide reliable measures.

### Differential item functioning

There was no evidence of DIF in any items in the scales according to gender, age, prior experience, entry qualification, or occasion of administration. Therefore, the scales can be accepted as representing the same construct for these different groups, and measures across different groups (for example, men and women, or the two occasions) may be legitimately compared.

### Targeting of items and persons

**Figure 5:**
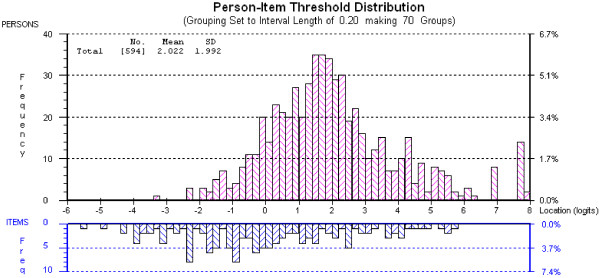
Distribution of person and item threshold locations for the PCCS.

**Figure 6:**
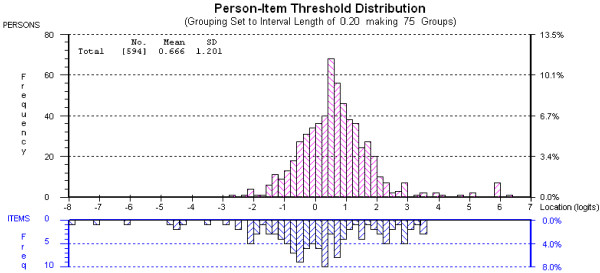
Distribution of person and item threshold locations for the CSCS.

Figure 5 and Figure 6, for the PCCS and CSCS, respectively, show person and item threshold location distributions relative to each other on a single continuum. Both scales could have more high-end items developed, in order to more reliably measure the most confident (highest scoring) students. However, the items (especially the CSCS) target the majority of participants well, that is, the items cover the same range on the continuum as the majority of the persons.

### Correlations with existing scales

Table 4 shows the correlation coefficients between scores on the two scales and the existing GSE and PRCA-24 (interpersonal communication) scales. As expected, the results showed a positive correlation between confidence scores for clinical and patient communication skills and general self-efficacy, and a negative correlation between confidence scores for patient communication and interpersonal communication (lower scores on the interpersonal communication scale represent less apprehension in communicating). The correlation between the PCCS and CSCS was quite high (0.686), indicating overlap between the two constructs represented by these scales, but a principal components analysis of the residual correlations when items from both scales were analysed together could not be carried out due to the absence of responses to blocks of items which were inappropriate for particular cohorts of participants. However, the lack of good fit when all items were analysed together, with many items from the CSCS misfitting, and the quite small number of item dependencies, suggests that the two scales are measuring relatively independent constructs. Further, they are constructs which it would be useful for clinicians to distinguish with different measures for their students.

**Table 4:** Correlations between new scales and existing scales

**Scale**	**CSCS**		**GSE**		**PRCA (sub-category)**	
	**r**	**probability**	**r**	**probability**	**r**	**probability**
PCCS	.686	p < .001	.378	p < .001	-.419	p < .001
CSCS			.241	p < .001	-.259	p < .001
GSE					-.343	p < .001

In summary, for the PCCS, all 28 items were retained with no alterations to response categories and a recommendation that item 20 be monitored in future research. The CSCS required a reduction in the number of response categories for two items, and a deletion of one item in order for the set of items to perform well as a scale to measure confidence in clinical skills. Additionally, it may be that the two scales could be combined into a single scale representing confidence in physical and person-oriented manual medicine skills. However, two measures are likely to be more useful than a single measure because they provide educators with a more focused guide when diagnosing where students most require help.

## Discussion

This paper has described the development and psychometric analysis of two new scales aimed at measuring confidence in clinical skills and in patient communication skills for students undertaking internships in manual medicine programs, particularly focussing on chiropractic students. The Rasch model has not previously been applied for psychometric analysis in the manual medicine field, and thus, in this regard, the study breaks new ground.

The first aim was to establish the internal consistency and reliability of the PCCS and CSCS. The results showed that the scales, with some amendments, provided internally consistent and reliable measures of confidence for participants. With the exception of very few items, items fitted the model. For example, in the PCCS, item 20 (*confidence in interviewing wheelchair bound patient*) was the least well-fitting. Whilst this item was intended to suggest a potentially difficult scenario and challenge students’ confidence, it did not discriminate amongst persons across the range of total scores and thus is likely to be assessing something other than the construct represented by the majority of the items in the scale. Further qualitative analysis is required to establish why it did not fit the model as well as the rest of the items in its scale, but it may be because it predicted a scenario which some students may and other students may not have encountered. Item 16 (*confidence in explaining segmental joint dysfunction*) also showed some misfit, but was retained at this stage. It’s content includes an important term in the chiropractic, physiotherapy and osteopathy professions, referring to a joint in the body not moving in a normal pattern. However, some chiropractic education programs may use the term ‘subluxation’ more often than ‘joint dysfunction’. Thus, for students unfamiliar with the term, the item measures knowledge of terminology. This highlights the need to carefully consider how each item is worded and whether the meaning mirrors the content of a particular program.

In the CSCS, item 1 (*overall confidence in clinical skills*) was the least well-fitting. The conclusion was that it was too broad in focus. This was also the case for item 1 in the PCCS, which measured overall level of confidence in patient communication and was amongst the easiest items for participants to agree with. Items that measure confidence in a general way should be reconsidered. In the CSCS, item 13 (*confidence in applying bracing/supports*) also showed some misfit. It tended not to discriminate as well as other items, possibly due to some students having more experience working in athletic and sporting areas. However, it was retained because it refers to an important competence in most chiropractic, physiotherapy, osteopathy and athletic training programs. Its performance could be monitored in future research.

High PSIs for the PCCS and CSCS provided statistical evidence of reliability. That is, the scales are able to discriminate amongst persons with different levels of confidence. However, both scales could benefit from items that require higher levels of confidence to endorse. For the PCCS, this might involve developing items that focus on being more supportive of patient needs, and on the handling of non-compliant patients, as these aspects were amongst the most difficult to endorse. For the CSCS, this might involve developing items that focus on manipulative skills with challenging patients since two items that required higher levels of confidence addressed working with a wheelchair-bound patient in one instance, and with a pregnant patient in the other instance.

During scale development, more items are usually created than are required to produce valid and reliable measures. Indeed, future use of these scales could involve item reduction, particularly reassessing the worth of items showing dependencies which indicate that one item in each pair is not adding significant information. For example, in the PCCS, six items deal with specific categories of people (gender, age and disability), and item dependencies indicate that only one of these items may be required to represent the concept of dealing with different categories of people.

The second aim was to verify whether each scale had similar psychometric properties across different groups of participants. There was no evidence of differential item functioning in the PCCS or CSCS according to gender, age, prior experience, entry qualification, or occasion of administration, which means that the scales can be accepted as representing the same construct across different categories of persons (for example, males and females for the gender group) within each of these groups. Therefore, measures on each scale may legitimately be compared across different sub-groups. This is particularly important when changes over time are being investigated or when different curricula for different groups of students are being considered.

The third aim was to investigate the convergent validity of the two new scales by examining the correlational association between the new scales and the established GSE and PRCA-24 (interpersonal communication) scales. Results showing a positive correlation between confidence scores for clinical and patient communication skills and general self-efficacy, and a negative correlation between confidence scores for patient communication and interpersonal communication, indicated significant, though relatively small, associations between the new and existing scales, and add to the evidence of the validity of the new scales. Hence the new and old scales measure similar constructs to some degree, but the indications are also that the new scales provide measures of distinctive properties.

In regard to validity, the Rasch analyses provide evidence of internal consistency which is an aspect of construct validity. Content validity was addressed by the assessment of a panel of educators and researchers, and convergent validity was assessed by the scores on the two new scales being correlated with scores from the same students on two existing scales. Consequential validity, a type of validity that addresses the intended and unintended consequences of test interpretation and use [51,52] may be assessed through examination of differential item functioning (DIF) or a close examination of the person-item map. These aspects provide information on the basis of which decisions for action are taken. Future studies may include principal component analysis of residuals and sub-scale analyses as a further check on the possible presence and usefulness of sub-scales within the PCCS and CSCS scales.

Limitations of the study include the focus on chiropractic students. The chiropractic field may not represent the full range of manual medicine programs, which also include osteopathy, physical therapy and athletic training fields. For example, osteopathy curricula can vary extensively, with many programs in the United States modelling medical education with minimal to no manual techniques, in comparison to programs in Australia, which have a strong focus on manual therapies. Physiotherapy (physical therapy) curricula especially emphasise pre- and post-surgical rehabilitation, and neuro-rehabilitation, such as post-stroke and post-spinal cord injury with chiropractic education covering this minimally. Athletic training specifically addresses on-field emergency procedures and pre-event functions such as taping, stretching of athletes and monitoring warm-ups. These noted differences in curricula, and thus professions, highlight the need to carefully consider the content of the items in the two new scales.

Another limitation is the analysis of the relationship between confidence and competence, which was not undertaken in this research. These constructs are intrinsically related, and arguably this relationship needs to be better understood, in order to recognise self-confidence as a vital construct which requires thorough investigation in health education. According to Bandura [6,15], the most effective way to acquire and enhance self-efficacy (closely associated with self-confidence) is through mastering skills. Future research would need to examine this relationship with the view to identifying the strength of correlations.

## Conclusion

This is the first study to undertake rigorous development and psychometric analysis of two new scales that can be applied during student internships in manual medicine programs. The results indicate that the two scales can provide educators and researchers with sound measures of student confidence in clinical and patient communication skills, which may then be used to identify students who require additional help, and to guide curriculum development. The scales may also be used to measure changes in levels of confidence in specific skills over time, and to examine the degree of congruence between confidence and competence.

## Competing interests

There are no financial or non-financial competing interests (political, personal, religious, ideological, academic, intellectual, commercial or any other) to declare in relation to this manuscript.

## Authors’ contributions

MH developed the scales. IS performed the content analysis. SV assisted with content analysis and outline of the project and paper. All authors contributed substantially to the conception and design of the study, as well as to the critical revision of the paper. All authors approved the final manuscript.
